# Convergent Validity of Step Counts Collected from a Smart Knee Implant and a Smartphone-Based Care Management Application: A 7861-Patient Study

**DOI:** 10.3390/s26031033

**Published:** 2026-02-05

**Authors:** Jason Cholewa, Karl Surmacz, Roberta E. Redfern, Mike B. Anderson, Krishna Tripuraneni, Nicola S. Piuzzi

**Affiliations:** 1 Zimmer Biomet, 1800 W. Center St., Warsaw, IN 46580, USA; karl.surmacz@zimmerbiomet.com (K.S.); roberta.redfern@zimmerbiomet.com (R.E.R.); mike.anderson@zimmerbiomet.com (M.B.A.); 2 New Mexico Orthopaedic Associates, 2100 Blvd NE, Suite 410, Albuquerque, NM 87110, USA; krishtrip@gmail.com; 3 Cleveland Clinic, 9500 Euclid Avenue, Cleveland, OH 44195, USA; piuzzin@ccf.org

**Keywords:** total knee arthroplasty, digital health monitoring, post-operative monitoring

## Abstract

Introduction: Step counts are increasingly used to assess mobility and track recovery following total knee arthroplasty (TKA). The purpose of this study was to assess the convergent validity of step count data captured by a smart implantable device (SID) in comparison with step counts derived from established, validated sensor-based technology. Methods: A secondary analysis of an anonymized commercial database (N = 7861, median age: 68, female: 59%, median BMI: 31.7) of patients who received an SID and used a digital care management application (App) with or without a smart watch. The SID recorded “qualified steps”, defined as periods of walking for at least seven steps that met predefined acceleration and cadence thresholds between 7 am and 10 pm. The App collected total daily step counts via smartwatch and/or smartphone. Pearson correlations were calculated between SID and App data at 30, 90, and 180 days post-operative. Step counts at 30, 90, and 180 days post-operative were compared between groups with the Mann–Whitney U test. Statistical significance was assessed at *p* < 0.001. Results: Step counts increased throughout the recovery period as measured by all three devices. SID-captured fewer qualified steps than App-captured step counts from watch-wearers throughout the post-operative period (*p* ≤ 0.001). SID step counts were similar to App step counts at 30 days post-operative and greater than App step counts at 90 and 180 days post-operative (*p* < 0.001). There were significant (*p* < 0.001), moderate correlations (r = 0.62 to r = 0.74) between step counts collected by the SID and App for both watch-wearers and smartphone-carriers at 30, 90, and 180 days post-operative. Conclusions: The SID’s qualified step metric demonstrated consistent, moderate, correlations with app-based step counts across 30, 90, and 180 days. While smartwatch-based tools recorded higher absolute step counts, both technologies reflected similar recovery trajectories.

## 1. Introduction

As healthcare increasingly shifts toward value-based care, the ability to measure objective functional outcomes following total knee arthroplasty (TKA) has become essential for guiding clinical decision-making, optimizing recovery, and demonstrating treatment effectiveness [1,2]. As such, the use of step counts to assess mobility recovery following TKA is increasing in popularity [3,4,5,6,7,8,9,10]. The first six-months post-operative appears to be a critical period whereby ongoing support and monitoring of patient activity may promote effective recovery [7,11]. Christensen et al. [6] have reported that, along with traditional patient-reported outcome scores, step counts demonstrated earlier improvements compared to other physical activity metrics. As the majority of activity recovery appears to occur within the first three months post-operative [12,13], the ability to monitor patient activity may also be used to promote appropriate return to function and pain management strategies [14].

Smart implant devices (SIDs) containing inertial measurement unit sensors (IMU) provide information on the mechanical aspects of daily knee function, including step counts. Because SIDs do not rely on patient compliance with external devices, they may allow for improved data capture rates compared to wearable technologies [9]. Further, the SID has been shown to consistently transmit qualified step counts post-operatively [13].

The data collected by SIDs may be used to facilitate personalized rehabilitation [9,15]. Previous studies suggest objective functional measures collected with a novel SID may be useful in understanding the potential for early intervention [16,17,18], and when assessed alongside patient-reported outcome measures (PROMs), may help to better understand patient recovery [13,19]. However, validation of the step count measurements with the SID has not been previously reported. The purpose of this study was to assess the convergent validity of step count data captured by a SID in comparison with step counts derived from established, validated smartphone and smartwatch sensor-based technology.

## 2. Materials and Methods

This was an IRB approved (WCG IRB# 20222582), non-randomized, secondary analysis of all patients (N = 7861) in an anonymized commercial database who received a smart knee implant (Persona IQ^®^, Zimmer Biomet, Warsaw, IN, USA) and were also using a smartphone-based care management application (mymobility^®^ Care Management Platform [App], Zimmer Biomet, Warsaw, IN, USA) with or without a smartwatch (Apple, Cupertino, CA, USA). The App uses data collected via Apple HealthKit^®^ (Apple Inc., Cupertino, CA, USA). All data was de-identified before review. The median age of the population was 68 years, and the majority were female (Table 1).

The SID (Canturio™ Tibial Extension (CTE), Canary Medical Inc., Carlsbad, CA, USA) contains an accelerometer, which collects ‘Qualified Steps’ [12]. The purpose of the qualified steps metric is to measure purposeful walking: periods of walking for at least seven steps, with no more than 1.5 s between steps, that meet a minimum acceleration and cadence threshold between 7 am and 10 pm. These thresholds are proprietary but intended to ensure only intentional walking sessions, as opposed to more random motion, are recorded. Data from the SID is transmitted nightly.

Similarly to the smart implant, the App collects total daily step counts via an accelerometer within the smart watch and/or the smart phone. Previous validation studies have demonstrated excellent correlation between App-derived step counts with pressure mat readings (r^2^ = 0.98) [20] and video assessment (r = 0.96) [21] of step counts in healthy participants. Recovery curves for qualified steps, steps collected via the App for smartphone-only users, and steps collected via the App for smartwatch wearers were calculated from day zero through 180 days post-operative based on a backwards-looking rolling seven-day mean, where the largest 5 data points are used within each 7 day window to remove outliers.

### Statistical Analysis

Pearson correlations were calculated between smart implant and App data at 30, 90 and 180 days post-operative. Pearson correlation coefficients were interpreted as 0.00 to 0.20: slight correlation, negligible relationship; 0.20 to 0.40: low correlation, definite but small relationship; 0.40 to 0.70: moderate correlation, substantial relationship; 0.70 to 0.90: high correlation, marked relationship; 0.90 to 1.00: very high correlation, very dependable relationship [22]. Bland–Altman analysis was used to assess agreement between SID- and App-derived step counts, complementing correlation-based analyses of convergent validity. Step counts at 30, 90, and 180 days post-operative were compared between groups with the Mann–Whitney U test. Statistical significance was accepted at *p* < 0.001.

## 3. Results

**Figure 1:**
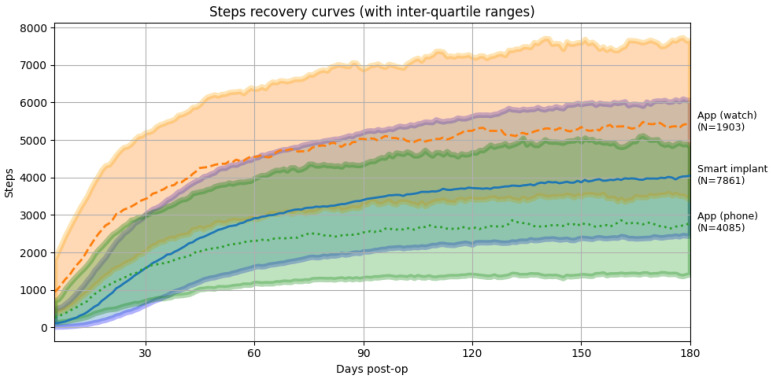
Step count recovery curve for smart implant (qualified step counts), watch-wearing, and non-watch wearing patients. The blue solid line represents the Smart Implant median; the blue shaded areas represent Smart Implant 25th and 75th percentiles. The green dotted line represents the phone median; the green shaded area represents the 25th and 75th percentiles. The orange dotted line represents the watch median; the orange shaded area represents the watch 25th and 75th percentiles.

All step count measures showed an increase in step counts throughout the recovery period (Figure 1). SID-derived step counts were consistently lower than App-based counts from smartwatch users throughout the post-operative period (*p* < 0.001, Figure 1). App-derived counts from smartwatch users were significantly higher than those from smartphone-only users (*p* < 0.001). SID step counts closely matched smartphone-only App data at 30 days (difference in medians of 3 steps) and exceeded them at 90 and 180 days postoperative (*p* < 0.001, Table 2). Interestingly, median SID-captured step counts continue to increase up to 180 post-operative days, whereas the App captured step counts plateau around 100 days post-operative.

**Figure 2:**
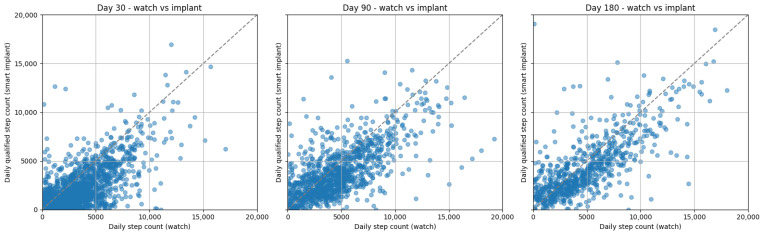
Step count correlations between the watch and smart implant at 30, 90, and 180 days post-operative.

**Figure 3:**
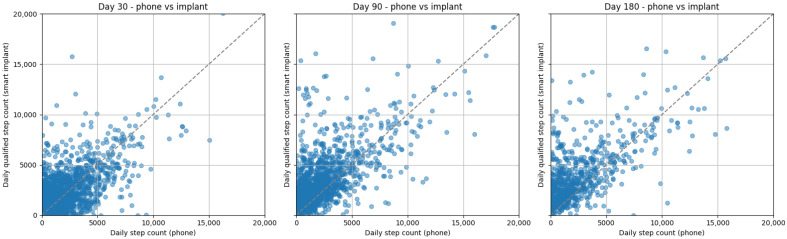
Step count correlations between the phone and smart implant at 30, 90, and 180 days post-operative.

**Figure 4:**
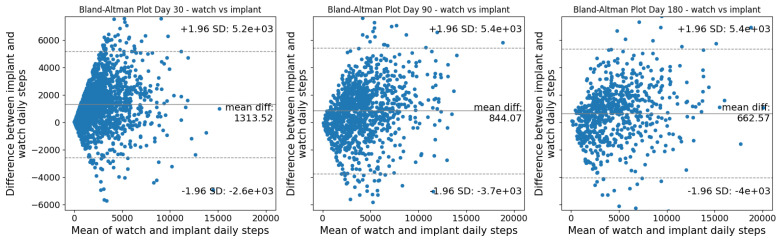
Bland–Altman plot between the watch and smart implant at 30, 90, and 180 days post-operative.

**Figure 5:**
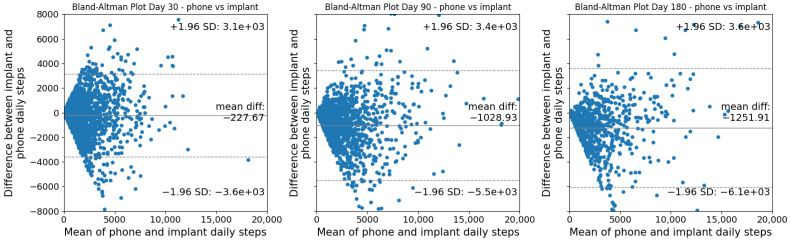
Bland–Altman plot between the phone and smart implant at 30, 90, and 180 days post-operative.

There were significant (*p* < 0.001), moderate correlations between step counts collected by the SID and App for both watch wearers and non-watch wearers at 30 (watch wearers: r = 0.66, non-watch wearers: r = 0.65), 90 (watch wearers: r = 0.68, non-watch wearers: r = 0.66), and 180 days (watch wearers: r = 0.74, non-watch wearers: r = 0.67) post-operative (Figure 2 and Figure 3). Bland–Altman plots for the watch and smartphone are presented in Figure 4 and Figure 5.

## 4. Discussion

This study demonstrated moderate correlative convergence between qualified step counts collected by the SID and step counts collected by the App, with correlations between r = 0.65 and r = 0.74 [22]. As expected, both qualified step counts and step counts measured via the App all increased during the immediate post-operative period, suggesting that all measures can be used to evaluate mobility recovery post-operatively following TKA, to some extent. These findings are consistent with those reported by Twiggs et al. [23] who concluded that step counts could be used to evaluate immediate post-operative recovery following TKA. They also align with findings from Pasqualini et al., who demonstrated that wearable step counts within the first 90 days post-TKA correlate meaningfully with patient-reported outcomes, reinforcing the role of real-world mobility metrics in evaluating recovery [11]. Moreover, the number of qualified steps measured in this study using the SID is consistent with prior reports [9].

The differences in qualified step count collected by the SID and step count collected by the App are predominantly due to differences in step collection methods between devices. While the App collects all steps as long as the patient is carrying their phone or using a wearable device, the SID only collects steps when a series of at least seven consecutive steps are taken above a threshold speed and cadence, deemed purposeful walking. Thus, there were likely periods of small movements or slow speeds, where the App was collecting step counts, but the SID was not. This is especially apparent early in recovery when slow, shuffling steps or the use of walking aids may not have achieved purposeful walking speed and cadence thresholds, thus accounting for the larger differences between devices.

On the one hand, this may represent an underestimation of step count by the SID early in recovery compared to the watch-derived step counts. On the other hand, Yocum et al. [19] suggests that qualified step counts representative of gait-based activity may provide a better evaluation of recovery compared to step counts from incidental activity. Lastly, the SID only collects steps between 7am and 10pm, whereas the App collects steps at all hours throughout the day. While this may capture activity in the majority of TKA patients, and the SID’s ability to passively and continuously monitor mobility without requiring patient adherence to external wearables offers a meaningful advantage in real-world care environments, the time window is a notable limitation in early risers or late exercisers that surgeons should be aware of.

The correlation between non-watch wearers and the SID was slightly lower at all time periods compared to watch wearers. Additionally, non-watch wearers either had fewer step counts collected or walked less compared to the SID and watch wearers, especially after 30 days post-operative. Smartphones demonstrate considerably greater mean absolute percentage errors than smartwatches [24] and have been shown to underestimate step count by 12% in free living conditions [25]. These discrepancies are most attributable to differences in device placement and user behavior. Smartwatches are worn on the wrists, which may provide more consistent step count capture compared to smartphones that are often carried in pockets or bags, or not carried at all, resulting in step count underestimation [25,26]. Regardless, it has been suggested that step counts measured by App users likely represent similar patterns in pre and post-operative user behaviors in relation to how they carry their phone and wear their watches [5].

The continued increase in median step counts captured by the SID through 180 days post-operative, compared with the earlier plateau observed in App-derived step counts, may be attributable to the App’s continuous walking and cadence-based detection criteria. Despite differences in absolute step counts, visual inspection of recovery curves and Bland–Altman plots demonstrates that both the SID and App reflect similar recovery trajectories, supporting the convergent validity of the SID. Correlations between the SID and App were modestly lower at 30 days post-operative for both watch wearers and non-watch wearers, but improved at 90 and 180 days, with Bland–Altman plots indicating increasing convergence by 180 days post-operative with lower levels of agreement in measures early on. Collectively, these findings suggest that while each measurement approach provides valid insight into post-operative recovery, absolute step counts are not directly interchangeable across instruments. Therefore, recovery should be assessed using consistent instrumentation, and surgeons should avoid switching between measurement modalities when tracking patient progress over time.

### Limitations

While this study is strengthened by its pragmatic design, large real-world sample, and data collected during free-living conditions, it is not without limitations. First, although the App shows excellent validity in healthy participants, caution should be taken when interpreting the data, especially in the first 30 days post-operative, as there is a paucity of literature validating the App in arthroplasty patients [27]. Secondly, the use of walking aids and slow shuffling steps in the first few weeks following TKA may impact data collection [27,28].

## 5. Conclusions

Overall, a substantial relationship in step count was found between the smart implant device and steps collected via a patient care management application, across early and mid-phase recovery milestones. These findings demonstrate the smart implant as a clinically meaningful, passive monitoring tool for tracking recovery after TKA. These results demonstrate similar patterns in recovery with convergence of the instruments, suggesting that the SID may be used as a tool for tracking mobility recovery after TKA and highlight its role in enhancing data-driven postoperative care; however, prospective interventional studies are necessary to confirm further utilities. As healthcare shifts toward outcome-driven and real-world data integration, smart implant technology offers a promising approach to extend orthopedic care beyond the isolated surgical event and into the recovery continuum.

## Figures and Tables

**Table 1:** Patient characteristics.

Age, median (25th, 75th percentile)	68 (62, 74)
Sex, n (%)	Female: 4525 (57.5%) Male: 3202 (40.7%) Not specified: 134 (1.7%)
BMI, median (25th, 75th percentile)	31.8 (27.0, 35.7)

**Table 2:** Differences in median step counts at 30, 90, and 180 days post-operative; *p* values represent significance assessment for a distribution comparison.

Comparison	Day 30	Day 30 *p*-Value	Day 90	Day 90 *p*-Value	Day 180	Day 180 *p*-Value
Smart implant steps—App steps (watch wearers)	−1844	<0.001	−1628	<0.001	−1426	<0.001
Smart implant steps—App steps (non-watch wearers)	3	0.039	865	<0.001	1245	<0.001
App steps (watch wearer)—App steps (non-watch wearers)	1848	<0.001	2493	<0.001	2672	<0.001

## Data Availability

The original contributions presented in this study are included in the article. Further inquiries can be directed to the corresponding author.

## Abbreviations

The following abbreviations are used in this manuscript:

TKA	Total Knee Arthroplasty
SID	Smart Implantable Device
IMU	Inertial measurement unit sensor
PROMs	Patient reported outcome measures
